# Inhalation of Sulphuric Ether to Prevent Pain during Surgical Operations

**Published:** 1847-04

**Authors:** 


### RECORD OF MEDICAL SCIENCE.

*Inhalation of Sulphuric Ether to prevent pain during Surgical operations.*—The following is a digest of the discussions of the learned societies of Paris concerning the properties of this agent, and of hospital reports, contained in the *Gazette des Hôpitaux* for the last month:—

*Académie de Medécine, Meeting, Jan. 12th,* 1847.—M. Malgaigne announced that he had tried upon five patients the method proposed by the Americans, to render surgical operations painless. The first patient was a young man, aged eighteen, who was afflicted with an abscess at the lower part of the leg. He breathed the ether for two minutes, which sufficed to plunge him into a state of complete lethargy. The abscess was opened with a bistoury. In half a minute afterwards, the patient woke up, and affirmed that he had experienced no pain, and up to that time believed he had not undergone any operation, but wished it to be proceeded with.

The second, an Italian, a little older, who had a tumour in the neck, respired the ether for five minutes. By the time he had revived, the operation was finished. He said he felt that the tumour had been removed, but had not experienced any pain.

The third patient was a young woman, also having a tumour in the neck. She did not fall into a state of insensibility until she had inhaled the vapour for eighteen minutes. She did not feel the first incision; but woke up immediately afterwards, and suffered during the rest of the operation as though she had not been submitted to its influence.

The fourth was a man who had had his leg broken by a rail-way truck, and underwent amputation at his own desire. He was submitted to the vapour of ether for seventeen minutes. On his coming-to, he said he had felt the operation, but had not suffered more pain than if he had lightly scratched his leg with the point of a knife.

The last, a young man, who was operated upon for strabismus, previously respired the ether for ten minutes without effect, and suffered during the operation as other patients would have done. In answer to a question from M. Nacquart, M. Malgaigne explained the process as used by the American surgeons, which he had adopted for the first patient; but for the others he had introduced into one of the nostrils, the other being closed, a tube leading from a vessel, the bottom of which was covered with ether—the patient inspiring by the nose, and expiring by the mouth.

M. Velpeau questioned whether ether was altogether innocuous to the system. He feared it might produce some injurious effect upon the patient, counterbalancing the advantage derived from the absence of pain. Besides, as the influence only lasted for a short time, its use in operations of a long duration was doubtful.

M. Guibourt had no fear of a bad result from the employment of ether, his only anxiety was as to the certainty of its operation, he himself having frequently and for a long time inhaled air strongly charged with ether, without experiencing any ill effect; and on this point he was supported by M. Chevallier.

M. Roux, of the Hôtel Dieu, detailed the particulars of a case of compound fracture of the leg in the *Gazette des Hôpitaux*, January 16th, 1847. The patient who was about forty-five years old, breathed by the mouth the vapour for twenty-minutes with great earnestness; in about ten minutes his eyes closed, but he still answered any questions put to him, and in ten minutes more the operation was finished; the pain thereof being evidently diminished, as the patient was not aware that the operation had been completed, until he was told such was the case.

*Hôpital St. Louis.*—A patient of M. Malgaigne—a man about thirty-five years old, of strong constitution, presented at the lower and internal part of the leg, about the level of the malleolus, a phlegmonous abscess—was submitted to the influence of the etherial vapour for two or three minutes, which short space of time sufficed to put him into the state necessary for the commencement of the operation, which can only be compared to drunkenness. M. Malgaigne addressed the patient, asking him whether he felt any particular sensation, or found his sight confused. The man having answered that his vision was imperfect, M. Malgaigne immediately used the bistoury, making an incision in the abscess and in a portion of the skin much supplied with nerve and considerably inflamed. He then pressed the pus from the abscess. On the termination of the operation the patient appeared agitated—his face was red, his features contracted, his eyelids closed, and, in short, his whole muscles, particularly those of the face and superior-extremities, exhibited symptoms of abnormal contraction. He seemed to be under the weight of painful feelings which he wished to shake off. He had undoubtedly lost his reasoning power, for his conduct was outrageous—he closed his eyes, and foamed from the mouth. This state lasted but two or three minutes. He was aroused by the voice of M. Malgaigne, and on recovering his consciousness he declared the pain was slight, not more than a prick, but he directly afterwards complained of the smarting which resulted from the wound.

A second patient, a man aged forty-five, had necrosis of the bones of the finger, resulting from a whitlow. M. Malgaigne determined to remove the finger, at the articulation, and caused the patient to inhale the ether for about four minutes. The patient declared he was drunk and unable to see. The operation was performed in the usual manner. This patient also compared the pain he felt to a prick, afterwards experiencing the sensation of smarting over the surface of the wound. The pulse was carefully observed during the operation, and found to be eighty-eight during the inhalation of the ether, and ninety-two after the operation.

A young girl of eighteen, with an affection of the hand, requiring incisions, was submitted to the action of the vapour, and in four minutes she declared her sight to be confused. She compared the operation to a prick. It is remarkable that in this case there was a considerable want of sensibility in the wound for some time after the operation.

M. Malgaigne administered wine in each of the last cases, to effect a more speedy recovery from the stupor; and it may be worthy of notice that the air exhaled was impregnated strongly with ether, so that it was impossible to mistake the agent that had been employed.

At the meeting of the *Académie de Medécine*, on Jan. 19th, a letter was read from M. Menière, respecting the successful employment of vapour of ether in cases of nervous deafness, hemicrania, paralysis of the facial nerve, complaints of the cavity of the cranium, &c.

M. Honoré mentioned a case of intense neuralgia, which was alleviated by the breathing of the vapour of ether, placed in a vessel with a large mouth, held close under the mouth.

M. Malgaigne made the following important observations as to the consequence of the use of ether:—In the case of amputation of the leg, he believed that there was less reaction than in ordinary cases; and another point, which he recommended to the attention of psychologists, was, that in most cases it appeared that the seat of sensation for pain was different from the seat of ordinary sensibility. Many patients retained perfect consciousness, understanding what was said to them, answering correctly, but feeling no pain; it really appeared to him that there were two centres of sensation.

A discussion also took place at the meeting of the *Academy of Sciences*, on January 18th, when M. Velpeau stated that he had failed in obtaining a complete and satisfactory result from the use of the vapour of ether. One patient had proved unmanageable. In another, the sensorial functions were evidently disturbed; but he had suffered pain while being operated on. A third had suffered in a like manner; but declared that he was plunged into such a state of ecstasy, that he was unable to complain. In short, it appeared that it succeeded with certain persons, and failed with others; and that it was not proved to be altogether without danger.

M. Dunos said that he had been led, from some experiments, to believe that the ether possessed a cataleptic power.

On the 22d of January, M. Velpeau, at the *Hôpital du Charité*, having used an apparatus constructed by M. Charrière, succeeded perfectly in removing a tumour without pain. The etherial vapour was inhaled by the patient about four minutes, after which time complete insensibility and relaxation of the muscles were manifested.

M. H. Larrey, who assisted at the operation, suggested the valuable assistance that the agent would render in the reducing of difficult luxations.

At the *Hôpital du Midi*, a case occurred in which the sensibility seemed to have been exalted by the inhalation.

The influence of the vapour has also been tested by M. Guersant, at the *Hôpital des Enfants*, on two children. One child, whose finger was amputated, declared that she felt pain, but was totally unable to cry out. The other, on recovering from the state of insensibility into which she had been thrown, declared that she had no recollection whatever of the operation.

At the *Hôpital du Midi*, M. Ricord, in injecting in a double hydrocele, employed the inhalation with success, though he was obliged to renew its influence twice during the operation. A second patient, afflicted with single hydrocele, after respiring vapour for thirteen minutes, fell into a complete state of insensibility; the limbs were relaxed; the pupils contracted; the conjunctiva was injected; and the pulse not affected. A third patient, who was apparently perfectly under the influence of the ether, suffered the usual amount of pain during an operation for removing a tumour from the rectum.

In the first two cases, a state of intense exhilaration preceded that of insensibility. In the last, the use of the ether was followed by sickness and fainting.

At the meeting of the *Societé de Chirurgie de Paris*, Jan. 13, 1847, M. Malgaigne mentioned a case in which the inhalation having been continued for too long a time, caused sinking of the pulse and coldness of the extremities to such an extent, that fears were entertained for the life of the patient.

At the meeting of the *Académie de Medécine*, Jan. 26, 1847, M. Landouzy called attention to a case, where hæmorrhage, after removal of a small tumour from the mastoid process, did not come on till half an hour after the performance of the operation; and suggested that surgeons should be on their guard lest accidents might happen from the arteries not being secured.

M. Honoré stated, that he had succeeded in relieving a patient afflicted with most obstinate neuralgia of the face, by the inhalation of ether vapour for about two minutes.

*Académie des Sciences*, Meeting, Jan. 25.—M. Gerdy related several experiments with the vapour of ether, the results of which had been satisfactory in the highest degree.

From the above it will be seen, that the success which our neighbours have met with has been varied; but we think that in most of the cases in which the ether failed to produce its stupifying effect, that fault was clearly in the instrument used for its administration. At first they attempted to use this agent by causing the patient to inspire by the nostrils, and respire by the mouth, and *vice versâ;* but afterwards they found it requisite to close the nostrils while respiration was carried on by the mouth alone.

The fact announced by M. Malgaigne, that some of the patients retained their consciousness, but felt no pain while being operated on, is most interesting, and we leave it to be commented on by physiologists; but the statement requires confirmation by other observers.

The influence, also, over the power of *expression* of pain, is also very curious, but seemed to be quite an uncommon result; for we only find that this was the case in two out of the numerous cases quoted.

The continued lethargy, with failing of the pulse, and coldness of the extremities, is certainly a most awkward complication to deal with in treating the shock of an operation, and one which should make us cautious in the employment of the vapour of ether. In this country, in more cases than one, this unpleasant effect followed its use.

If the vapour of ether prove an efficient therapeutic agent in the treament of neuralgic affections, then, indeed, will its introduction prove a boon to society. And we much regret that M. Honoré did not give a more detailed account of those cases in which he employed this remedy.—*London Lancet.*

